# Ultrasound-guided platelet-rich plasma injection for traumatic painful neuroma of brachial plexus: a case report and literature review

**DOI:** 10.3389/fneur.2025.1546773

**Published:** 2025-03-17

**Authors:** Haifeng Zhu, Zixuan Deng, Yongqi Xie, Guifeng Qian, Danyu Wang, Shaodong Xie, Yunyi Zhang, Peichun Yan

**Affiliations:** ^1^Department of Rehabilitation Medicine, Foshan Hospital of Traditional Chinese Medicine, Foshan, China; ^2^The Eighth Clinical Medical College, Guangzhou University of Chinese Medicine, Guangzhou, China; ^3^Department of Ultrasound Diagnosis and Treatment, Foshan Hospital of Traditional Chinese Medicine, Foshan, China

**Keywords:** brachial plexus, neuroma, neuropathic pain, platelet-rich plasma, ultrasonography, ultrasound guided injection

## Abstract

Traumatic neuroma (TN) is a repair response of nerves to direct/indirect trauma or chronic inflammatory injury, commonly occurring after trauma or surgery. The authors report a rare case of a traumatic painful neuroma of the brachial plexus. Physical therapy and drug treatment failed to resolve the symptoms of allodynia and a palpable mass, which significantly reduced the patient’s quality. Ultrasound-guided injection of platelet-rich plasma (PRP) has shown significant efficacy in repairing the nerve and relieving pain. However, there is a lack of research on treating TN with PRP injection. This case demonstrates that ultrasound-guided injection of PRP can alleviate neuropathic pain caused by a traumatic painful neuroma of brachial plexus and improve the upper limb function.

## 1 Introduction

Traumatic neuromas (TNs) form in nerves during repair and regeneration following direct or indirect trauma, chronic inflammatory injury, or surgery, and are usually caused by abnormal proliferation of nerves, excessive scar formation, or excessive distal severing of neural tissue as a reparative response to nerve injury (1, 2). Histopathologically, neuromas usually present as a significant thickening of the epineurium with incomplete replacement of nerve bundles (nerve fascicles), forming an abnormal mixed structure of Schwann cells, fibroblasts and collagen fibers (3). This structure lacks the classical hierarchical compartmentalisation of the nerve bundles and instead forms a distinct histological compartment within the common nerve sheath. Immunohistochemical stains specific for Schwann cells (S-100) and vessels/fibroblasts (CD34) can be used to accurately identify the aforementioned histopathological changes (4). Clinically, the most common symptoms of TN include sensory abnormalities such as pain, numbness, discomfort, or electric shock-like sensations. Approximately 20–30 percent of neuromas are painful, significantly impairing the patient’s quality of life (5, 6). However, most conservative treatments only provide temporary pain relief to patients with limited therapeutic effect (7). Platelet-rich plasma (PRP) therapy utilizes platelet-derived growth factors to promote the three phases of wound healing: inflammation, proliferation, and remodeling (8). PRP represents a promising advancement in tissue regeneration and analgesia (8–10). There is a paucity of studies on the use of platelet-rich plasma injections for treating traumatic painful neuromas. The purpose of this article is to present a case of ultrasound-guided PRP injection for treating a traumatic painful neuroma of the brachial plexus and to review the relevant literature.

## 2 Case presentation

**Figure 1:**
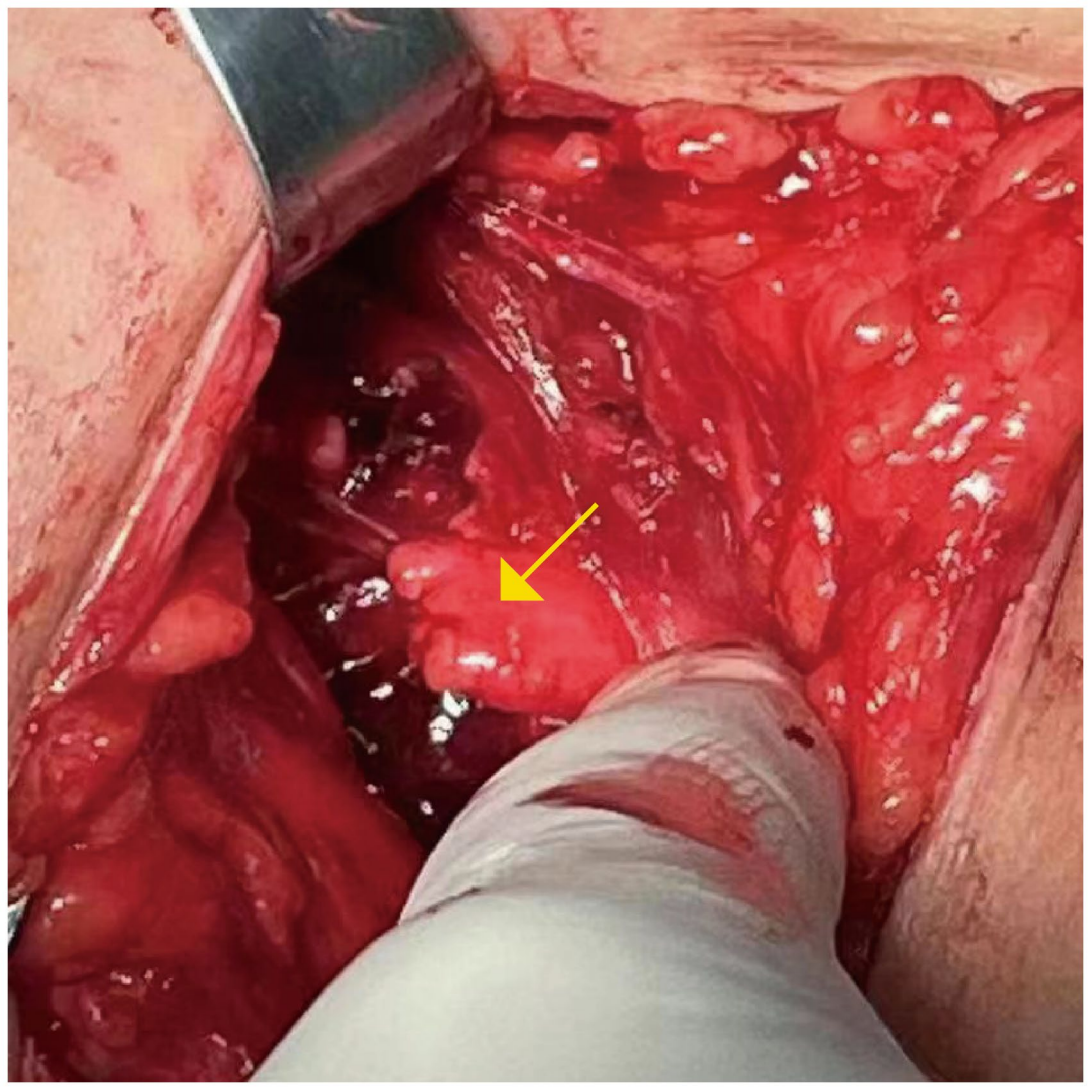
The brachial plexus nerve anastomosis (C5-C6) was performed at a local hospital. The nerve rupture is marked with a yellow arrow.

A 31-year-old male suffered from a sharp instrument injury to his left shoulder on December 5, 2022. As a result, his left upper limb experienced pain, weakness, numbness, and restricted movement. Following admission, examination revealed brachial plexus nerve damage, and brachial plexus nerve anastomosis (C5-C6) was performed at a local hospital on December 5, 2022 (Figure 1). Although his left upper limb motor function improved and discomfort was reduced following surgery, the patient’s quality of life remained significantly affected by left shoulder pain. After discharge, the patient still experienced pain and sought outpatient treatment. He was prescribed 300 mg of gabapentin and 50 mg of chlorpromazine hydrochloride tablets daily. Subsequently, the efficacy of the medication diminished, and increasing the dosage failed to adequately control the pain. On March 6, 2023, the patient developed left upper limb motor and sensory disturbances, accompanied by stabbing pain and intermittent electric shock-like sensations. The pain worsened at night and was exacerbated by passive movement. The patient sought treatment at the Department of Rehabilitation Medicine, Foshan Traditional Chinese Medicine Hospital. Since the onset of the condition, the patient has experienced spontaneous episodes of depression, anxiety, and poor sleep quality. There was no significant medical history, personal history, or family history. This case report was approved by the Ethics Committee of our hospital (approval number: KY-2024-323).

### 2.1 Examination

Significant muscle atrophy was observed in the left shoulder, with proximal muscle strength graded as level 2 and distal muscle strength as level 4. The left shoulder joint exhibited flexion of 30°, extension of 30°, and abduction of 20°. And the left elbow joint showed extension of 0° and flexion of 45°. No significant abnormalities were observed in the range of motion (ROM) of the left wrist, palm, and finger joints. Decreased skin sensation was noted in the left upper limb. Tenderness was present in the left shoulder, and the Tinel sign was positive at the surgical scar. The visual analog scale (VAS) score was 6 points, the Disability in Arms, Shoulders, and Hands (DASH) score was 55 points, and the Pittsburgh Sleep Quality Index (PSQI) score was 18 points (Table 1).

**Table 1:** Comparison of pain, sleep, and upper limb function scores before and after treatment.

	Before treatment	The second PRP treatment	The sixth PRP treatment	Follow up after half a year
VAS	8	5	2	0
PSQI	19	13	7	4
DASH	66.6	41.3	17.5	0

PRP, Platelet-Rich Plasma; VAS, Visual Analog Scale; PSQI, the Pittsburgh Sleep Quality Index score; DASH, the Disability in Arms, Shoulders, and Hands score.

**Figure 2:**
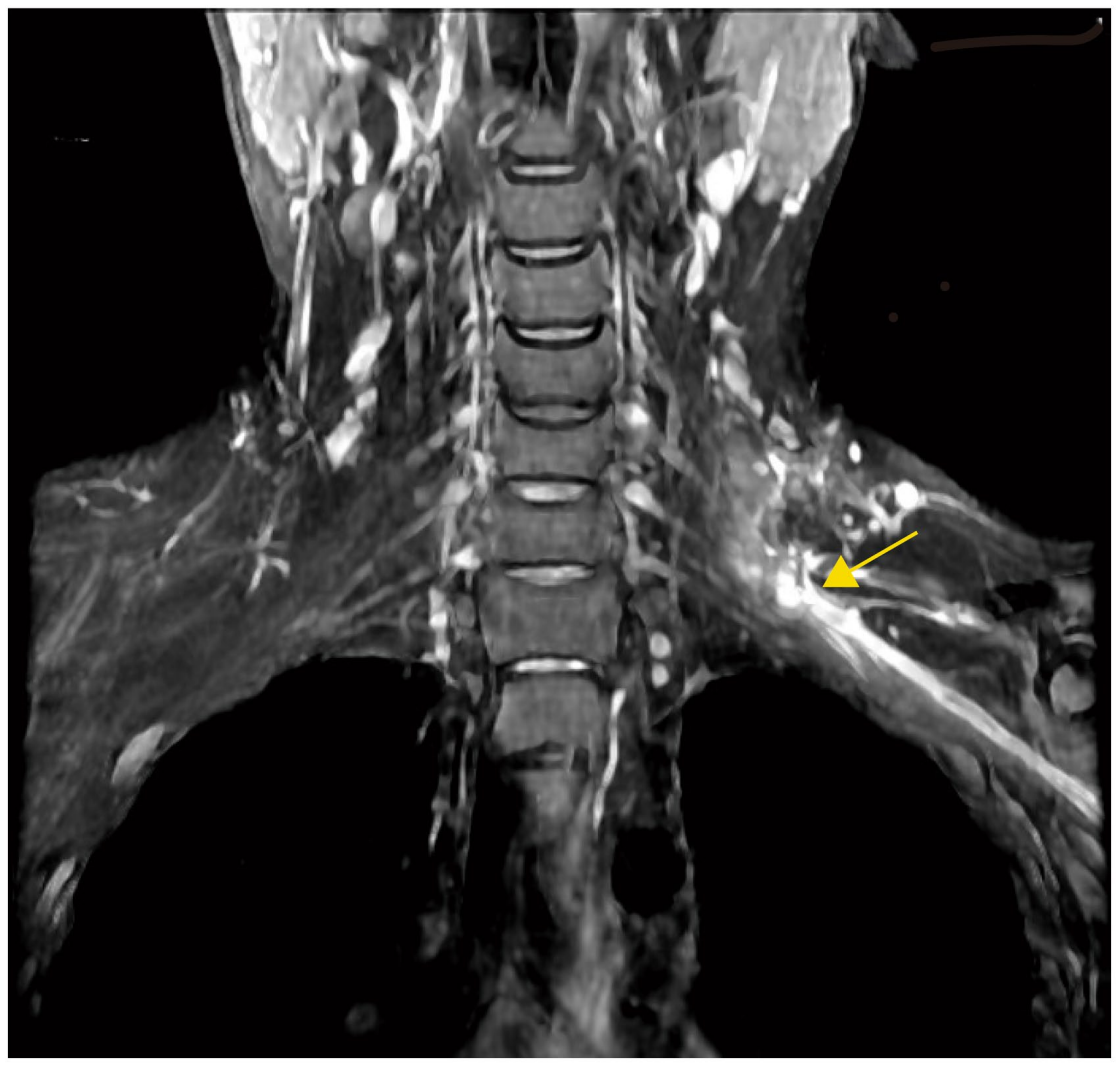
MRI image for the treatment of brachial plexus neuroma. The location of the neuroma after neuroanastomosis is marked with a yellow arrow.

**Figure 3:**
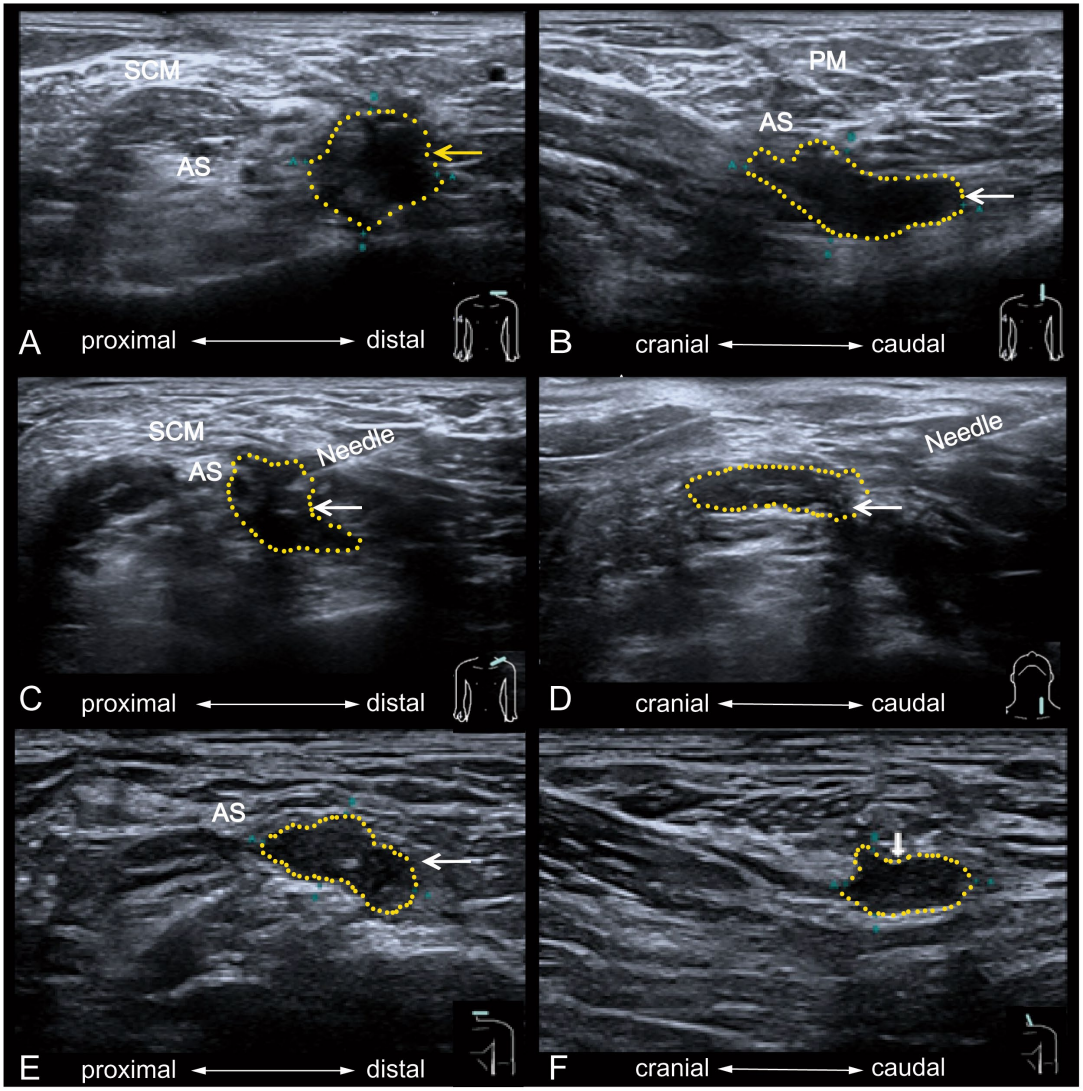
Ultrasound imaging before and after PRP injection treatment for brachial plexus neuroma. The left brachial plexus neuroma is seen in its short-axis **(A)** and long-axis **(B)** before the treatment. It shows a lack of a distinct envelope in the area of the left brachial plexus trunk lesion, the presence of irregularities, and structural disorganization of the nerve fibers. After ultrasound-guided injection in the brachial plexus neuroma is seen in short-axis **(C)** and longaxis **(D)**. The short-axis **(E)** and long–axis **(F)** shows the image of the left brachial plexus neuroma after 6 months, and the neuroma decreases in tissue size. PM, platysma myoides. SCM, sternocleidomastoid muscle. AS, anterior scalene muscle. The yellow dashed area encircles the brachial plexus neuroma.

MRI of the brachial plexus revealed post-injury changed on the left side, showing the anterior branch of cervical nerves 5 to thoracic 1 with a continuous course and local appearance resembling small cystic lesions (Figure 2). Ultrasonographic examination revealed the formation of a traumatic neuroma in the upper branch of the left brachial plexus at the clavicular level (Table 2), measuring approximately 14 mm × 7 mm × 13 mm (Figure 3). Electromyography (EMG) revealed neurogenic damage in the left biceps brachii, triceps brachii, flexor carpi ulnaris, flexor carpi radialis, deltoid, and infraspinatus muscles. The upper trunk of the left brachial plexus was severely damaged, while the middle trunk and medial bundle showed mild damage.

**Table 2:** Comparison of the size of brachial plexus traumatic neuroma between the first and last treatment under ultrasound.

		Before treatment	The sixth PRP treatment
The short axis	Distant A	147.0 mm	14.0 mm
	Distant B	138.0 mm	6.8 mm
The long axis	Distant A	237.0 mm	11.1 mm
	Distant B	97.0 mm	5.6 mm

PRP, Platelet-Rich Plasma.

### 2.2 Treatment

Based on the patient’s physical examination and ancillary tests, the diagnosis was traumatic painful neuroma of the left brachial plexus. During hospitalization, the treatment plan included medication and physical therapy. Drug therapy included mecobalamin (0.5 mg, three times daily) and pregabalin (150 mg, twice daily). The physical therapist performed joint mobilization of the shoulder, including flexion, abduction, and internal and external rotation, for 20 min per session, twice daily. Resistance strength training was also performed for the right biceps brachii, triceps brachii, and deltoid muscles for 20 min per session, twice daily. Physical agent therapies, including mediumfrequency electrical stimulation, neuromuscular electrical stimulation, and infrared therapy, were administered for 20 min per session. However, despite conventional treatment, the patient continued to report pain and developed symptoms of depression and anxiety.

After discussing the risks and benefits of PRP injection, the patient provided written informed consent and received six ultrasound-guided platelet-rich plasma injections. Ultrasound imaging of the brachial plexus is challenging due to its complex neural network. Ultrasound-guided injections were performed by a sonographer, as described in reference (11). The PRP preparation process was as follows: (1) 16 mL of whole blood was drawn from the patient’s median cubital vein and evenly divided into two separate tubes containing 3.8% w/v sodium citrate, with each tube containing 8 mL. (2) The whole blood was centrifuged. (3) After removing two-thirds of the platelet-poor plasma by gravity for 5 min, the remaining one-third was collected by gently shaking the tube, yielding 4 mL of PRP. The patient was placed in a supine position, with the pillow removed and the head slightly tilted backward. The surgeon applied pressure to the surgical scar, eliciting electric shocklike pain in the patient’s left arm to identify the puncture site. After disinfection and local anesthesia, the operator held a 5 cm, 7-gage puncture needle in their right hand and inserted it vertically. Ultrasound guidance was used to target the upper branch of the left brachial plexus. After confirming the needle position and ensuring no aspiration of blood, fluid, or air, approximately 4 mL of plateletrich plasma was injected around the nerve. Ultrasound imaging confirmed PRP infiltration around the nerve (Figure 3), with no complications observed during the 30-min monitoring period. The entire procedure was performed under sterile conditions, with treatments administered over 6 weeks and injections given every 2 weeks.

### 2.3 PRP treatment results and follow-up

The patient received six PRP injections between March 9, 2023, and May 23, 2023. Following the first PRP injection, the patient reported reduced severity and frequency of nighttime pain, and pain relief was noted during passive joint exercises. After the sixth treatment, the patient’s pain score, PSQI score, and DASH score were significantly lower than pre-treatment values (Table 1).

Ultrasound imaging revealed a reduction in the size of the traumatic neuroma (Table 2). The patient was able to resume normal daily activities and work. During the six-month follow-up after the final treatment, the patient reported no pain or discomfort during daily activities, improved sleep quality, restored upper limb function, and discontinuation of oral medications. EMG examination revealed significant improvement in the motor fibers of the upper trunk of the left brachial plexus, with recovery observed in the middle trunk and medial bundle compared to pre-treatment findings. However, no significant changes were observed in the sensory fibers.

## 3 Discussion

The patient in this case had a traumatic neuroma of the brachial plexus, and the patient’s pain was poorly controlled with conventional medications and rehabilitation. Ultrasound-guided injection of PRP reduced the patient’s pain, relieved anxiety, and also promoted the recovery of the patient’s upper limb function through subsequent rehabilitation. The results of this case indicate that ultrasound-guided injection of PRP for treating brachial plexus traumatic neuroma has a good analgesic effect. No adverse events were observed during the treatment and follow-up period, which is of some clinical reference significance.

### 3.1 Pain mechanisms of traumatic painful neuroma

The most common site of TN is the lower limb after amputation, followed by the head and neck, with other sites including the radial nerve and brachial plexus (12). TN in the brachial plexus is an extremely rare condition with few reports in the literature. Traumatic neuroma is a proliferative and reparative neurological response, often presenting as a nodular mass with clinical manifestations of pain hypersensitivity and resistance to most analgesics (13). And painful TN resistant to most analgesics. The mechanisms of pain associated with TN are complex and may involve neuropathic pain-related pathophysiological changes (14). Mechanisms associated with nociceptive neuroma pain include: (1) Local inflammatory reactions caused by peripheral nerve injury (15). (2) Peripheral nerve injury may lead to changes in the sympathetic nervous system and sensitization of both the peripheral and central nervous systems, resulting in abnormal pain. (3) Proliferation of myofibroblasts wrapping around axons, which induces neuroma pain through local contraction of *α*-SMA (16). (4) A significant increase in unmyelinated fibers in neuromas, which are abnormally proportioned to myelinated fibers, increases the sensitivity of nerve fibers to mechanical stimuli (15, 17).

### 3.2 Treatment of traumatic painful neuroma

There is no standard treatment for TN. Patients with TN who are suitable for surgery have significant pain relief after surgical intervention, but there is no uniform, effective treatment plan for patients who do not meet the indications for surgery or who are unwilling to undergo surgical treatment (18). Different conservative treatments have positive therapeutic effects on different areas of TN, such as physiotherapy, local injections (lidocaine or alcohol), cryotherapy, radiofrequency ablation, shockwaves, and electrostimulation (13, 19, 20). Currently, transcutaneous electrical nerve stimulation is more commonly used in physical therapy for neuropathological pain (21), and transcranial magnetic stimulation (TMS) is also applied to alleviate neuropathic pain (22). A study evaluating the role of HF-rTMS and tDCS in pathologic pain after brachial plexus injury found that pain was significantly reduced in the treatment group and persisted for at least 1 month (23). However, fewer literature reports on PRP for the treatment of brachial plexus neuromas.

### 3.3 Mechanisms of PRP in the treatment of neuromas

PRP is a product derived from the patient’s own blood, containing a variety of growth factors, such as platelet-derived factor, vascular endothelial growth factor, and transforming growth factor (8, 24, 25). PRP is widely used in clinical settings for tissue regeneration and repair. Due to its theoretical potential to repair tissues with low regenerative capacity and its apparent safety, platelet-rich plasma is becoming increasingly popular for treating pain associated with muscle injuries, osteoarthritis, and other conditions (26–28).

However, the mechanism of action for PRP in alleviating neuropathic pain remains unclear. Existing clinical studies primarily focus on the anti-inflammatory and analgesic effects of PRP, which are mediated by the release of cytokines that regulate the local microenvironment (29). Studies investigating the anti-inflammatory effects of PRP have significantly alleviated ectopic pain in a rat model of neuropathic pain induced by burns over a four-week period. Simultaneously, it reduced the expression of spinal cord cytokines (e.g., CCL2) and downregulated the p38 mitogen-activated protein kinase signaling pathway. These findings suggest that PRP might influence neuropathic pain by modulating inflammation and nerve growth factor expression (30). Patients with peripheral neuropathic pain can undergo direct PRP application to damaged nerves. Research by Hassanien et al. revealed that, compared to the control group, peripheral injection of PRP improved pain and neuropathic scores in patients with diabetic neuropathic pain for at least 6 months (31). A study by Dong et al. found that PRP treatment was advantageous for pain relief and nerve regeneration in patients with mild to moderate carpal tunnel syndrome (32).

### 3.4 Limitations

Although it is known that PRP contains various growth factors and cytokines, such as plateletderived growth factor and transforming growth factor-*β*, which play significant roles in the tissue repair process. However, the specific mechanisms of PRP in the treatment of traumatic painful neuromas remain only partially clear. For instance, how these growth factors precisely regulate the proliferation, differentiation, and migration of nerve cells, as well as their interactions with the surrounding neural microenvironment, has not yet been fully elucidated. This lack of clarity in the mechanisms limits the further optimization and precise application of PRP treatment protocols.

## 4 Conclusion

This case report is the first to document the improvement of neuropathic pain in traumatic neuromas through ultrasound-guided PRP injection, offering a novel approach to treating traumatic painful neuromas. However, the mechanisms underlying PRP treatment for neuropathic pain and nerve repair remain unclear. Single-case reports have limitations. Future research on PRP treatment for painful neuromas should explore inclusion and exclusion criteria, treatment methods, follow-up outcomes, and confounding variables to provide higher levels of evidence-based medicine for PRP treatment of painful neuromas.

## Data Availability

The raw data supporting the conclusions of this article will be made available by the authors, without undue reservation.
